# High-Quality Draft Genome Sequences of Four Lignocellulose-Degrading Bacteria Isolated from Puerto Rican Forest Soil: *Gordonia* sp., *Paenibacillus* sp., *Variovorax* sp., and *Vogesella* sp.

**DOI:** 10.1128/genomeA.00300-17

**Published:** 2017-05-04

**Authors:** Hannah L. Woo, Kristen M. DeAngelis, Hazuki Teshima, Karen Davenport, Hajnalka Daligault, Tracy Erkkila, Lynne Goodwin, Wei Gu, Chien-Chi Lo, Christine Munk, Matthew Scholz, Yan Xu, Patrick Chain, David Bruce, Chris Detter, Roxanne Tapia, Cliff Han, Blake A. Simmons, Terry C. Hazen

**Affiliations:** a Joint Bioenergy Institute, Emeryville, California, USA; b Department of Civil and Environmental Engineering, University of Tennessee, Knoxville, Tennessee, USA; c Microbiology Department, University of Massachusetts–Amherst, Amherst, Massachusetts, USA; d Bioscience Division, Los Alamos National Laboratory, Los Alamos, New Mexico, USA; e Department of Microbiology, University of Tennessee, Knoxville, Tennessee, USA; f Department of Earth and Planetary Sciences, University of Tennessee, Knoxville, Tennessee, USA; g Institute for Secure and Sustainable Environment, University of Tennessee, Knoxville, Tennessee, USA

## Abstract

Here, we report the high-quality draft genome sequences of four phylogenetically diverse lignocellulose-degrading bacteria isolated from tropical soil (*Gordonia* sp., *Paenibacillus* sp., *Variovorax* sp., and *Vogesella* sp.) to elucidate the genetic basis of their ability to degrade lignocellulose. These isolates may provide novel enzymes for biofuel production.

## GENOME ANNOUNCEMENT

Previous studies have shown that plant litter decomposition can occur rapidly in tropical forests (1). The strains presented here were cultivated under oxic conditions from the soil of the Luqillo Experimental Forest in Puerto Rico using lignin or cellulose in minimal media agar (2). These strains were selected for genome sequencing based on their ability to degrade model carbohydrates or phenolics (2).

The genomes were sequenced by the Joint Genome Institute using Illumina sequencing technology. The short and long paired-end library preparation and assembly followed the methodology previously described by Everroad et al. (3). To raise the quality of the final sequence, PCR PacBio consensus sequences were used to close gaps. The total amount of data from the Illumina reads ranged from 3.9 to 5.7 Mb per isolate. The average G+C content was between 50 and 67%. *Variovorax* sp. had the largest estimated genome at 7.7 Mb. The genome sizes of *Gordonia* sp. and *Paenibacillus* sp. were fairly similar at about 6.9 Mb and 6.3 Mb, respectively. *Vogesella* sp. had the smallest genome size at 4.2 Mb (Table 1).

**TABLE 1:** Metadata of Puerto Rican soil strains and sequencing run details

Species	Isolation carbon source	Strain	Phylum; order	Genome size (Mb)	G+C content (%)	Accession no.	Sequencing library paired-end reads (short/long)	Total reads (Mb)	Coverage
*Gordonia* sp.	Alkali lignin	HW436	*Actinomycetes*; *Actinomycetales*	6.3	67	ARVZ00000000	14,821,334/19,192,428	4,142	657×
*Paenibacillus* sp.	Carboxymethyl cellulose	HW567	*Firmicutes*; *Bacillales*	6.9	50	ARFI00000000	14,326,726/18,199,996	4,879	707×
*Vogesella* sp.^a^	Alkali lignin	LIG4	*Proteobacteria*; *Neisseriales*	4.2	64	LT607802	18,542,852/11,702,786	3,952	941×
*Variovorax* sp.	Alkali lignin	HW608	*Proteobacteria*; *Burkholderiales*	7.7	67	LT607803	14,536,906/35,495,686	5,730	744×

a Originally submitted as *Pseudogulbankiania* sp. LIG4.

The genomes possess genes related to lignocellulose degradation. Genomes of three bacterial strains isolated on alkali lignin (*Variovorax* sp., *Gordonia* sp., and *Vogesella* sp.) possess the beta-ketoadipate pathway for aromatic catabolism of lignin monomers and other phenolics into tricarboxylic acid cycle intermediates (4). *Variovorax* sp. and *Gordonia* sp. have multiple dioxygenases to metabolize two different aromatic catabolism intermediates (protocatechuate and catechol), while *Vogesella* sp. only has genes for protocatechuate degradation. *Paenibacillus* sp., the strain isolated on carboxymethyl cellulose in minimal media, possesses five different endo-1,4-betaxylanses. One or more of these xylanases could be highly active, as *Paenibacillus* sp. grows well on cellulose and xylan agar and degrades beta-d-glucopyranoside at notable rates.

*Variovorax* sp. and *Vogesella* sp. may also contribute to nitrogen cycling. *Variovorax* sp. has nitrogenases (*nifK*, *nifD*, and *nifH*) that are related to nitrogen fixation. *Vogesella* sp. has genes encoding respiratory nitrate reductase (alpha, beta, and gamma subunits) that are related to dissimilatory nitrate reduction.

All four genomes are part of an ongoing investigation of the genetic basis of lignocellulose degradation in tropical soils. These genomes will be compared to other genomes of lignocellulose-degrading bacteria from tropical forest environments, such as *Enterobacter lignolyticus* SCF1 (5), *Klebsiella* sp. BRL6-2 (6), and *Burkholderia* sp. LIG30 (7). The discovery of genes encoding lignocellulose-degrading enzymes would benefit biofuel production, for which lignocellulosic biomasses must be rapidly deconstructed and saccharified using enzymes.

### Accession number(s).

The whole-genome sequences reported here were deposited in DDBJ/EMBL/GenBank under the accession numbers listed in Table 1. *Vogesella* sp. LIG4 was originally submitted in 2012 as *Pseudogulbankiania* sp. LIG4, another *Neisserales* species, but has since been determined to be more closely related to other *Vogesella* sp. strains by 16S rRNA gene analyses with BLASTn and average nucleotide identity by BLAST (ANIb).
